# Satellite Imaging and Long-Term Mosquito Surveillance Implicate the Influence of Rapid Urbanization on *Culex* Vector Populations

**DOI:** 10.3390/insects10090269

**Published:** 2019-08-24

**Authors:** Eleanor N. Field, Ryan E. Tokarz, Ryan C. Smith

**Affiliations:** Department of Entomology, Iowa State University, Ames, IA 50011, USA

**Keywords:** mosquito surveillance, mosquito ecology, urbanization, land use, *Culex*

## Abstract

The ecology and environmental conditions of a habitat have profound influences on mosquito population abundance. As a result, mosquito species vary in their associations with particular habitat types, yet long-term studies showing how mosquito populations shift in a changing ecological landscape are lacking. To better understand how land use changes influence mosquito populations, we examined mosquito surveillance data over a thirty-four-year period for two contrasting sites in central Iowa. One site displayed increasing levels of urbanization over time and a dramatic decline in *Culex pipiens* group (an informal grouping of *Culex restuans,* *Culex pipiens,* and *Culex salinarius*, referred to as CPG), the primary vectors of West Nile virus in central Iowa. Similar effects were also shown for other mosquito vector populations, yet the abundance of *Aedes vexans* remained constant during the study period. This is in contrast to a second site, which reflected an established urban landscape. At this location, there were no significant changes in land use and CPG populations remained constant. Climate data (temperature, total precipitation) were compiled for each location to see if these changes could account for altered population dynamics, but neither significantly influence CPG abundance at the respective site locations. Taken together, our data suggest that increased landscape development can have negative impacts on *Culex* vector populations, and we argue that long-term surveillance paired with satellite imagery analysis are useful methods for measuring the impacts of rapid human development on mosquito vector communities. As a result, we believe that land use changes can have important implications for mosquito management practices, population modeling, and disease transmission dynamics.

## 1. Introduction

Mosquitoes (Diptera: Culicidae) are highly diverse, with 3565 species documented in temperate and tropical climates around the world [1]. Although occupying a wide range of environments, all mosquitoes require suitable aquatic habitats for their immature stages. Depending on the mosquito species, larval habitats vary from natural to artificial that include: standing water, water-accumulating plants (i.e., bromeliads), artificial receptacles, brackish marshes, tree holes, and even hoof prints [2]. In each of these microhabitats, detritus and decaying matter serve as nutritional resources for developing larvae [3], while adults seek out nectar or take a blood meal to provide resources for egg production [4].

The success of a mosquito species in a given location is invariably tied to its ability to find acceptable larval habitats, energy sources, resting habitat, and acceptable hosts for blood–feeding. Urban, semi-urban, and rural environments provide different challenges and environmental features for organisms to navigate. Some insect species readily adapt to close human proximity, while others prefer rural locations [5]. Highly urban environments are often associated with decreased species abundance and species richness [6]. With 68% of the human population expected to live in urban areas by 2050 [7], increased urbanization has the potential to significantly influence mosquito distributions and mosquito-borne disease transmission.

Urbanization has been described as a primary driver of mosquito population change in North America [8]. This includes decreases in mosquito abundance and increased species richness, where land use may be a more important factor than climate change in altering mosquito communities [8]. These changes in mosquito population dynamics as a result of urbanization can also have important implications for vector-borne disease transmission, causing an increase in some vector species such as *Aedes albopictus* and *Anopheles gambiae* [9,10].

In Iowa, 55 species of mosquitoes have been described [11], with the nuisance species *Aedes vexans* being the most abundant [12]. *Culex pipiens* group (CPG; a collective term encompassing the informal grouping of morphologically similar *Culex pipiens, Culex restuans,* and *Culex salinarius* suggested by Sucaet et al. [12]) are the second most abundant species in the state [12] and are the primary vectors of West Nile virus (WNV) transmission in central Iowa [13,14]. However, our understanding of how changes in land use influence these mosquito species is limited.

To examine how mosquito populations vary over time, we examined mosquito species dynamics at two locations in central Iowa over a 34-year study period. One site experienced increasing urbanization, while the other remained relatively unchanged. Mosquito populations changed significantly at the site with increased urbanization: where a massive decline in *Culex pipiens* group mosquitoes coincided with a period of sustained commercial and residential development. In contrast, no significant changes in land use or mosquito population structure were detected at the stable urban site during the extended study period. Together, these data suggest that habitat loss resulting from urbanization significantly alters CPG mosquito populations, which could have important implications for disease transmission.

## 2. Materials and Methods

### 2.1. Mosquito Trapping Locations and Sample Collections

**Figure 1:**
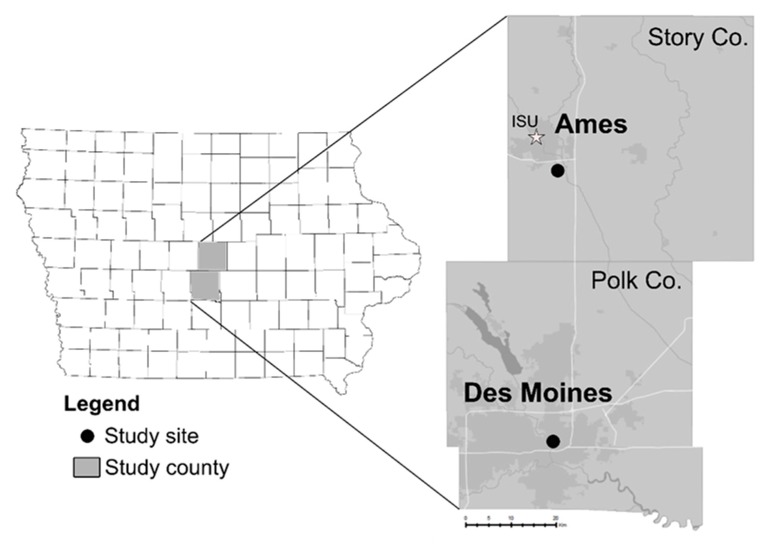
Overview of trapping locations in central Iowa. Historical mosquito abundance data were examined from study sites in two central Iowa counties (Story and Polk) over a 34-year period. The Story County site was located in Ames (urbanizing), near Iowa State University (star), while the Polk County site was located in Des Moines (established urban). For county maps, major highways (white lines), urban areas (medium gray shading), and rivers/lakes (dark grey) are denoted to display county features.

For two locations in central Iowa (Figure 1), historical data from single New Jersey light traps (NJLTs) were used to measure mosquito population counts at each site. The first trap was run from 1975–2008 in Ames, Iowa (42°00′08″ N 93°36′02″ W) representing an urbanizing environment, while the second trap was run from 1976–2009 in a public park in Des Moines, Iowa (41°36′42″ N 93°36′35″ W), an established urban landscape.

With some yearly variance, traps were run continuously from mid-May to early October each year. Samples were pooled by week, and identified to species using morphological keys [15]. To normalize trapping efforts for collection time differences between years, the trap index (total mosquitoes collected/number of active trap nights) was used rather than raw mosquito counts.

### 2.2. Remote Sensing of Trap Locations

Landsat 5 TM CM1 Level-1 imagery was obtained at five-year intervals from 1984–2004 using the public domain United States Geological Survey’s (USGS) EarthExplorer (https://earthexplorer.usgs.gov/). For each of the years utilized, high-quality images (less than 10% scene cloud cover and less than 10% land cloud cover) were selected from July and August to reflect the period with the highest historical mosquito abundance. All images were imported and edited using the clip tool in ArcGIS 10.4.1 software to convert from the full image file to an output extent of the study area. This area encompassed the study site as well as a buffer, reflecting a 4 km radius surrounding the NJLT centroid. This distance was selected based on a mark release program that found 82% of recaptured *Ae. vexans* specimens occurred within 4 km [16]. The flight distance for *Ae. vexans* was selected to set the radius, as they have a larger flight range than *Culex* species [17], and were a pre-dominant species at both locales. This distance would also account for any direct landscape changes that would influence mosquito abundance at the trapping site.

### 2.3. Land Use/Land Cover (LULC)

To examine land use changes in the study areas, land use/land cover models were generated using the Spatial Analyst extension in ArcGIS 10.4.1 software. A supervised classification of each Landsat raster image was conducted using the Maximum Likelihood Classification method. Five different land use classes (wetland/water, tree cover, barren, agricultural, and built) were defined from each image where applicable. Wetland/water refers to rivers and immediate floodplain areas, where tree cover was attributed to heavily forested areas. Barren land refers to unused, non-vegetative land (i.e., dirt). Lastly, the agricultural class identified agricultural areas in all stages of crop development, while the built class refers to all zones of development such as roads, commercial, industrial, and residential areas. Land usage was determined using the Create Signatures tool to generate a unique signature file, which was then input into the Maximum Likelihood Classification tool to generate the classified raster output. The pixel count of each land use classification was recorded from the attribute table of the newly created classified raster. The total number of pixels in each site was divided by the total number of pixels representing each land class type within that site, with the resulting outputs converted to a percentage and compared temporally to depict changes in land usage.

### 2.4. Environmental Variables

Climate data was collected using Iowa State University’s Environmental Mesonet database (https://mesonet.agron.iastate.edu/request/coop/fe.phtml) using the IA0200 station located in Ames for each year of the study period (1975–2008) and the IATDSM station in Des Moines for 1976–2009. Data was obtained from 1 May–1 October, which comprise the active trapping months of Iowa’s mosquito surveillance program. Total rainfall (mm) was recorded by adding the weekly precipitation counts within this time period. Temperature was measured using Growing degree-days determined from weekly averages of each year. Growing degree-days are a preferred measurement of temperature rather than Fahrenheit or Celsius for agricultural or insect development purposes [14,18,19,20]. Warm-season standards (temperature base 50 °F and max 86 °F) were used as thresholds for all analysis [18]. Growing degree-days were computed by the Mesonet database, but are calculated as follows: Degree-day accumulation = [(Maximum Temperature + Minimum Temperature)/2]—Base Temperature [18].

### 2.5. Statistical Analysis

Simple linear regression (SLR) was used to examine population and climatic changes over time using GraphPad Prism software. Through this approach, temperature as growing degree days and rainfall were examined for both study sites to determine potential changes during the study period. Additional SLR was performed to test the effects of land use percentage changes on CPG populations where land use percentages and CPG trap indices were examined at 5-year increments. Mann-Whitney analyses were performed in GraphPad Prism software to compare CPG populations at the Ames and Des Moines sites for the respective first- and second-half of trapping at each site.

## 3. Results

### 3.1. Analysis of Mosquito Population Dynamics over Three Decades in Central IOWA

**Figure 2:**
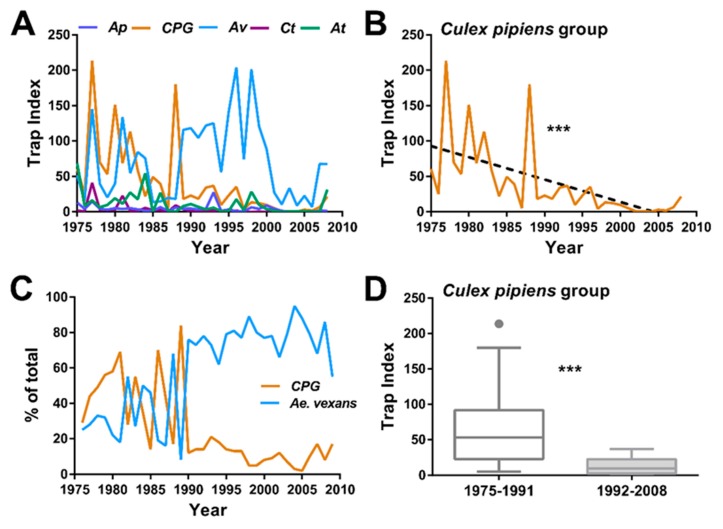
Temporal analysis of mosquito abundance from the Ames, Iowa trap site (1975–2008). Trap index averages (standardized abundance) for each year at the Ames site for the five most abundant mosquito species (**A**): *Anopheles punctipennis* (*Ap*), *Culex pipiens* group (CPG), *Aedes vexans* (*Av*), *Culex tarsalis* (*Ct*), and *Aedes triseriatus* (*At*). A significant decline in CPG populations were detected over the study period when analyzed by simple linear regression (R^2^ = 0.3719, *p* < 0.0001, F = 18.95, df: 1, 32) (**B**) The dynamics between the two most abundant species, *Ae. vexans* and CPG, are more apparent when examined by relative abundance where distinct differences in populations are observed sometime after 1990 (**C**). When CPG populations are compared between the first half of the trapping period (1975–1991) and the second half (1991–2008), there are significant differences in the overall trap index when analyzed using Mann-Whitney analysis (*p* < 0.0001, two-tailed, exact) (**D**).

A total of 490,568 mosquitoes were collected over 34 years from 1975–2008 at the trapping location in Ames, Iowa (Figure 1). Raw numbers were normalized by trapping effort to account for differences between years, and mosquito abundance data for the five most prevalent species were visualized using a standardized trap index (Figure 2A). When examined using simple linear regression, we recorded a highly significant decline in CPG (R^2^ = 0.3719, *p* < 0.0001, F = 18.95, df: 1, 32; Figure 2B). *Ae. triseriatus* and *Cx. tarsalis,* although not largely abundant, experience slight, yet significant population decreases (R^2^ = 0.1641, *p* < 0.05, F = 6.284, df: 1, 32; R^2^ = 0.1720, *p* < 0.05; F = 6.649, df: 1, 32, respectively; Figure S1). *Ae. vexans* and *An. punctipennis* do not experience population changes over the period (R^2^ < 0.001, *p* = 0.9719, F = 0.001, df: 1, 32; R^2^ = 0.1022, *p* = 0.0653, F = 3.643, df: 1, 32, respectively; Figure S1). Focusing on the relative abundance of the two most abundant species, *Ae. vexans* and CPG, we observe a dramatic decline in CPG numbers approximately midway through our study period (Figure 2C). To further demonstrate these differences, the study period was split in half, grouping the data into two equal trapping periods (1975–1991 and 1992–2008). CPG numbers were dramatically reduced in the 1992–2008 trapping period (*p* < 0.0001; Figure 2D), suggesting that these mosquito populations were negatively influenced by one or more external factors.

**Figure 3:**
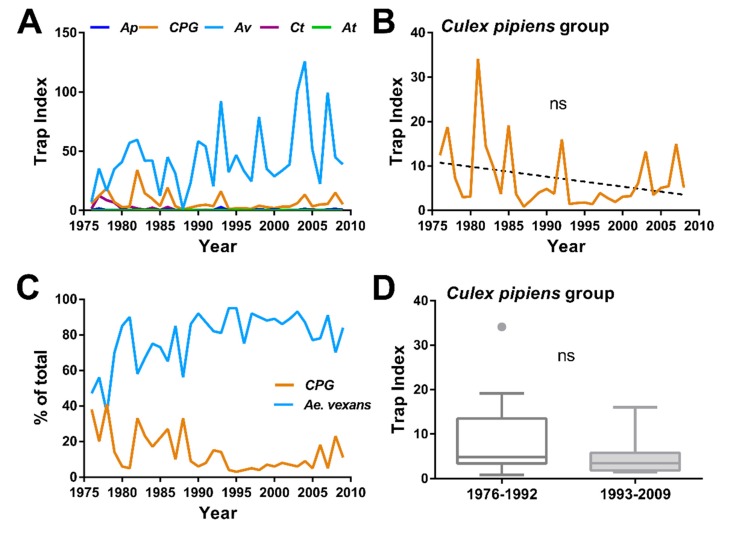
Temporal analysis of mosquito data from the Des Moines, Iowa trap site (1976–2009). Trap index averages (standardized counts) for each year at the Des Moines site for the five most collected mosquito species (**A**): *Anopheles punctipennis* (*Ap*), *Culex pipiens* group (CPG), *Aedes vexans* (*Av*), *Culex tarsalis* (*Ct*), and *Aedes triseriatus* (*At*). The relative abundance of the two most common species, *Ae. vexans* and CPG, are displayed over the study period (**B**). No significant differences (R^2^ = 0.07793, *p* = 0.1099, F = 2.704, df: 1, 32) were detected in *Culex pipiens* group (CPG) populations over time using simple linear regression (**C**) or when trap index averages were compared for the first half (1976–1992) and the second half (1993–2009) of the study using Mann-Whitney analysis (*p* = 0.1598, two-tailed, exact) (**D**).

To enable comparative analysis, we similarly examined mosquito population dynamics at a nearby site in Des Moines, Iowa (Figure 1), where a total of 165,694 mosquitoes were collected over an equivalent 34-year period (1976–2009). Combined trap indices revealed lower overall mosquito population numbers, and were dominated by *Ae. vexans* (Figure 3A). In contrast to the Ames site, CPG populations remained stable at the site in Des Moines (R^2^ = 0.07793, *p* = 0.1099, F = 2.704, df: 1, 32); Figure 3B). However, there was a significant increase in *Ae. vexans* (R^2^ = 0.1393, *p* < 0.05, F = 5.18, df: 1, 32; Figure S2) and a significant decrease in *Cx. tarsalis* populations (R^2^ = 0.3065, *p* < 0.001, F = 14.14, df: 1, 32; Figure S2), which are less prevalent. No changes recorded for *An. punctipennis* (R^2^ = 0.03070, *p* = 0.3216, F = 1.013, df: 1, 32; Figure S2), although this species was not commonly collected. No specific changes in the dynamics between *Ae. vexans* and CPG were detected over the study period (Figure 3C), or when the CPG trap index was compared between 1976–1992 and 1993–2009 (*p* = 0.1598, two-tailed, exact; Figure 3D). Together, these data suggest that mosquito populations were relatively stable at the Des Moines site, and that the reduction in CPG populations is unique to the Ames site.

### 3.2. Changes in Land Use Influence Mosquito Populations

**Figure 4:**
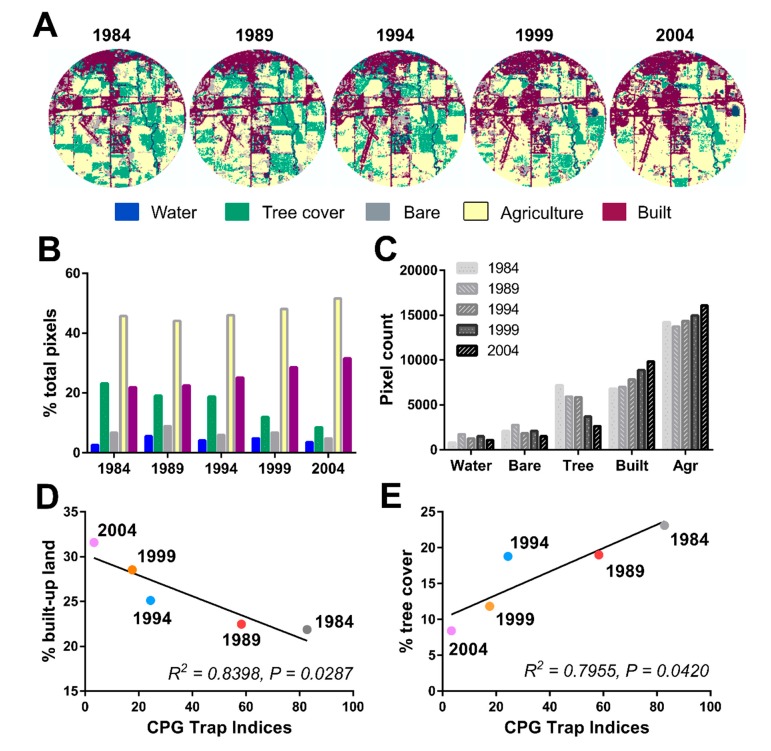
Changes in land use and land cover near the Ames site from 1984–2004. A supervised classification time series of land use/land cover every five years from 1984 to 2004 shows increases in built-up land over time (maroon) and a decrease in tree-covered land (green) in a 4 km fixed area around the Ames trap site (**A**). Changes in land use/land cover classification are displayed by each year according to classification (**B**) or by classification over time (**C**). Values are displayed as percentages of total pixels or total pixel count respectively. Relationships of CPG abundance (averaged over a five-year period ending in the year displayed) were examined by simple linear regression with the percentage of built-up land (**D**) or percent tree cover (**E**). Land use percentages for the 5-year intervals were derived from land use/land cover classifications above.

Since changes in land use could have profound influences on mosquito habitats, we examined satellite images from both site locations to determine how basic landscape characteristics have changed during the study period. Satellite imagery from 1984 to 2004 was used to examine changes in land use near the Ames trap site (Figure S3). To quantify these changes, a land use/land cover (LULC) classification was run on the satellite imagery using ArcGIS (Figure 4A). From 1984 to 2004, this analysis revealed incremental differences in built-up, agricultural, and tree cover over the twenty-year period (Figure 4B). This includes an overall increase in the percentage of agricultural land (12.9%) and built land (44.4%), while losses in wetland (32.4%), tree cover (63.6%), and bare land (27.3%) were detected (Figure 4C). These changes confirm that the area near the Ames trapping site underwent pronounced development over the study period.

To connect changes in landscape near the Ames trap site to the decline in CPG populations, a simple linear regression was run using the land classification percentages for built-up land and tree cover (Figure 4). Annual CPG populations were similarly grouped into five-year increments to match the five-year increments used for land use measurements. These results show a negative relationship between the percentage of built-up land with CPG trap index values (R^2^ = 0.8398, *p* = 0.0287, F = 15.72, df: 1, 3; Figure 4D) and a positive relationship between percent tree cover and CPG trap index values (R^2^ = 0.7955, *p* = 0.0420, F =11.67, df: 1, 3; Figure 4E). Similar analysis examining correlations of CPG populations with water, bare, or agricultural changes had no effect (Figure S4). Together, this suggests that specific changes in land use near the Ames site significantly impacted CPG populations.

**Figure 5:**
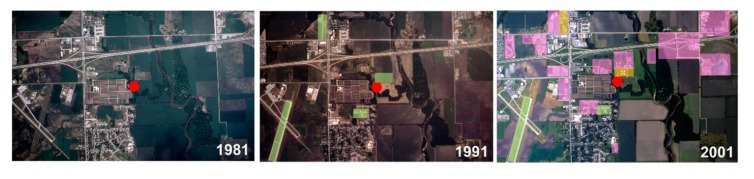
Specific land use modifications near the Ames site from 1981–2001. Satellite images at ten-year intervals from 1981 to 2001 were used to identify locations that underwent substantial changes in land use. The red dot denotes the trap location, while specific changes are displayed in green (occurring between 1981–1991), purple (occurring between 1991–2001), or yellow (occurring between 1991–2001 at a location that had previously been modified).

To better understand the specific changes that may have led to this significant decline in CPG populations, USDA flyover imagery was obtained of the Ames trapping site at ten-year intervals from 1981 to 2001 (Figure 5). During the period from 1981 to 1991, there were four discernable locations that underwent development. However, from 1991 to 2001, there were a total of 13 places that displayed substantial changes in land use, including several in close proximity to the Ames trapping site (Figure 5). While it is unlikely that a single modification caused the decline in CPG populations after 1991, the combined site modifications between 1991–2001 near the trap site suggest that the changes in land use may have extensively disrupted CPG habitats. If these changes occurred during a period of low numbers due to natural fluctuations in CPG abundance, these habitat changes could have created conditions where CPG numbers could not rebound as they did previously (Figure 2). In contrast, few changes occurred along the Skunk River and its adjoining flood plains (Figure 5), providing support that *Ae. vexans* populations would not be influenced by these same landscape modifications.

**Figure 6:**
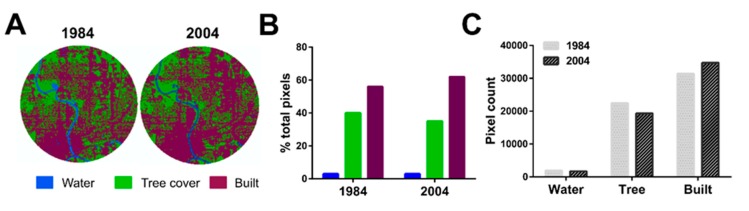
Changes in land use and land-cover near the Des Moines site from 1984–2004. A supervised classification of land use/land cover was performed for 1984 and 2004 to measure built-up land (maroon) and tree-covered land (green) over time using a 4km fixed area around the Des Moines trap site (**A**). Changes in land use/land cover classification are displayed by each year according to classification (**B**) or by classification over time (**C**). Values are displayed as percentages of total pixels or total pixel count respectively. Land use classifications falling under the *agriculture* or *bare* terms were not identified for the Des Moines site and were therefore excluded in the presentation of the data.

In contrast, satellite imagery from the site in Des Moines, from 1984 to 2004 (Figure S5) and subsequent LULC analysis did not reveal substantial changes in land use (Figure 6A). There is a slight increase in built-up land (10.6%) and a reciprocal loss of tree cover (13.9%) (Figure 6B), yet over the study period the majority of land use remained built (Figure 6C), reflective of an established suburban landscape. When paired with mosquito abundance data, the minimal changes to land use at the site are likely reflected by the stability of the CPG populations.

### 3.3. Examination of Climate Conditions over the Study Period

To examine if climate conditions were changing over the period and may have contributed to mosquito population changes, a simple linear regression of temperature (average Growing degree-day) and total rainfall (mm) for the mosquito trapping months (May–October) over the study period were performed. No significant changes were detected for either temperature (R^2^ = 0.003157, *p* = 0.7523, F = 0.1014, df: 1, 32) or seasonal rainfall (R^2^ = 0.04625, *p =* 0.2219, df: 1, 32) at the Ames location (Figure S6). These results are mirrored at the Des Moines site for both temperature (R^2^ = 0.01877, *p* = 0.4398, F = 0.6120, df: 1, 32) and total rainfall (R^2^ = 0.002512, *p* = 0.7783, F = 0.08060, df: 1, 32) (Figure S6).

To further investigate if climatic conditions could account for the changes in mosquito populations at the Ames site rather than land changes, a simple linear regression analysis was performed to determine the relationship of temperature and rainfall on CPG populations (Figure S7). No significant relationships were determined for temperature (R^2^ = 0.07676, *p* = 0.1127, F = 2.661, df: 1, 32) or total rainfall (R^2^ = 0.0057, *p* = 0.6713, F = 0.1834, df: 1, 32). These data provide further support that the decline in CPG populations at the Ames site (Figure 2) were not due to changes in climate, but rather due to landscape modifications.

## 4. Discussion

While landscape has been intrinsically tied to differences in mosquito ecology [17], there is a limited number of studies that have examined how habitat changes can influence mosquito population dynamics. Taking advantage of a long-standing mosquito surveillance program, we examined historical mosquito abundance records from two site locations, each with 34 years of continual trapping records. Using this valuable resource, mosquito abundance records were examined across the study period and compared. Although limited in scope, our robust study of two central Iowa locations revealed distinct differences in *Culex pipiens* group populations over time, likely influenced by landscape changes near the Ames trapping location.

A primary signature of urbanization is the growth of man-made substrates, increased human activity, and disturbance to existing habitat. The associated habitat fragmentation and loss of heterogeneity can select for certain mosquitoes, and decrease species abundance near the trapping locations [21,22]. We see similar patterns in our study for mosquito populations at the Ames site, where landscape changes have likely directly affected mosquito numbers by physically altering larval habitats and adult overwintering sites. However, additional indirect effects of urbanization from artificial lighting [23], pollution [24], noise [25], or increased mosquito control activities could have also influenced mosquito populations near the Ames trapping location.

Often, urbanization comes at the loss of natural, forested habitats. The loss of tree cover has been previously implicated as being an important aspect of urbanization effects on insect communities [26] and may have affected CPG mosquitoes in several ways. First, it serves an immediate purpose for *Culex* species as a resting habitat; these mosquitoes are highly active during the morning and evening, spending the day resting in protected areas of high humidity, of which tree cover provides [17]. Second, forested areas and their associated tree cover can serve as overwintering habitat for *Culex* [27]. Without a hibernaculum, the overwintering adult female mosquitoes are unable to survive the low temperatures which could reduce repopulation efforts for the following spring. While *Culex* species can overwinter in man-made structures [28,29], it is possible that the continued construction over several years near the Ames trap site would create disturbances for overwintering and nearby larval habitats. This is supported by the decline in *Ae. triseriatus* and *Cx. tarsalis* populations in addition to CPG at the Ames site, which respectfully utilize treeholes or other man-made structures for hibernacula [17]. This could also explain why *Ae. vexans* populations did not decline, as they overwinter in the protected egg state along riverbanks or flood plains, making them less vulnerable to the elements or the impacts of development [3,30].

A number of other factors may have contributed to this decline that can never be fully realized in a retrospective, historical analysis. One major limitation of this analysis is the inability to distinguish between *Culex* species within the CPG classification used for mosquito identification in our study. *Cx. pipiens, Cx. restuans,* and *Cx. salinarius* are identified morphologically using subtle features [15] that are often difficult to distinguish in mosquito samples collected from NJLTs [11,12,13,14,31]. However, other studies performed in close proximity to the trapping locations in central Iowa have suggested that *Cx. restuans* and *Cx. pipiens* respectfully comprise the vast majority of CPG mosquitoes collected the region [14,32]. Evidence suggests that both *Culex* species have similar breeding habitats [33], yet seasonal patterns driven by temperature influence their abundance [14,32]. Both *Cx. pipiens* and *Cx. restuans* are not known to be strong fliers, with their maximum flight range thought to be only 2.48 km and an average flight range of 1.15 km [34]. Thus, their ability to escape landscape alterations is hampered, along with their ability to fly to reach suitable nectar sources or find oviposition sites. This differs from some *Aedes* species, which have been shown to travel 40 m or more during their lifespan [17]. These differences could also explain why CPG populations underwent a dramatic decline in response to a changing landscape, whereas *Ae. vexans* were unaffected.

Climate has been previously implicated as having a strong effect on mosquito distribution [35]. There was a slight increase in total rainfall from 1975 to 2008, with 1993 being a notably higher-than-average year for summer precipitation corresponding with a massive, regional flood that occurred [36]. Yet, as a whole, total seasonal precipitation amounts did not significantly change over the 34-year period at either location. Rainfall and temperature have both specifically been noted as important factors of *Culex* population dynamics [14,37], yet we did not find significant relationships of population abundance with rainfall or temperature in our study. This is likely due to the use of seasonal rainfall and temperature measurements, where the influence of these environmental variables are likely less pronounced than on a smaller temporal scale. In addition, the population dynamics of *Cx. restuans* and *Cx. pipiens* are largely influenced by temperature, but can vary greatly temporally within a season or between years, while overall *Culex* numbers remain relatively constant [14]. From our analysis, it seems more likely that physical landscape changes were more important in driving the changes to CPG populations in our study although we cannot exclude that climate also had some influence on variation in mosquito numbers.

While we provide evidence that landscape changes influenced mosquito population dynamics near the Ames site, the exact year or impetus driving these changes is impossible to conclusively state from retrospective studies. Given the widespread landscape changes occurring between 1991 and 2001 near the site, the collapse in CPG, *Cx. tarsalis*, and *Ae. triseriatus* populations is likely due to multiple events that created ongoing habitat disturbances which negatively influenced mosquito populations. When paired with natural interannual variations in mosquito numbers, each habitat disturbance may have diversely impacted mosquito species where specific landscape alterations could influence certain mosquito species more than others.

## 5. Conclusions

In summary, we argue that landscape changes brought on by population growth and expansion were likely involved in the population decline of *Culex* vector populations in a central Iowa location. As a result, we speculate that these results will extend to other parts of the state and likely to other geographic regions, highlighting the importance of temporal studies on mosquito populations. However, due to the inherent differences in species composition, climate, and ecology, the influence of urbanization will likely not be uniform. Therefore, as human populations continue to grow and landscapes become more urbanized, there is a need for similar studies to better understand how human interactions influence mosquito populations. Since *Culex* species are primary vectors of West Nile in the United States, we believe that understanding the drivers that influence long-term mosquito distribution is crucial to our understanding of human disease risk.

## Supplementary Materials

The following are available online at https://www.mdpi.com/2075-4450/10/9/269/s1, Figure S1: Comparisons of Aedes vexans population abundance at two central Iowa study sites, Figure S2: Comparisons of mosquito population abundance at the Des Moines study site, Figure S3: Unaltered Landsat 5 imagery of the Ames trapping site at five-year increments from 1984–2004, Figure S4: Influence of land use variables on Culex pipiens group (CPG) populations at the Ames trapping site, Figure S5: Unaltered Landsat 5 imagery of the Des Moines trapping site, Figure S6: Climate variables do not significantly vary during the 34-year trapping period for both central Iowa sites, Figure S7: Culex pipiens group (CPG) abundance does not significantly correlate with temperature or rainfall.
