# Supplementary material for: Satellite Imaging and Long-Term Mosquito Surveillance Implicate the Influence of Rapid Urbanization on Culex Vector Populations
Source: Insects. 2019 Aug 24;10(9):269. doi: 10.3390/insects10090269 (PMC6780205; doi:10.3390/insects10090269)
Supplement: Supplementary file 1 [file insects-10-00269-s001.pdf]

Supplementary Materials

Satellite Imaging and Long-Term Mosquito Surveillance Implicate the Influence of Rapid Urbanization on *Culex* Vector Populations

Eleanor N. Field, Ryan E. Tokarz and Ryan C. Smith

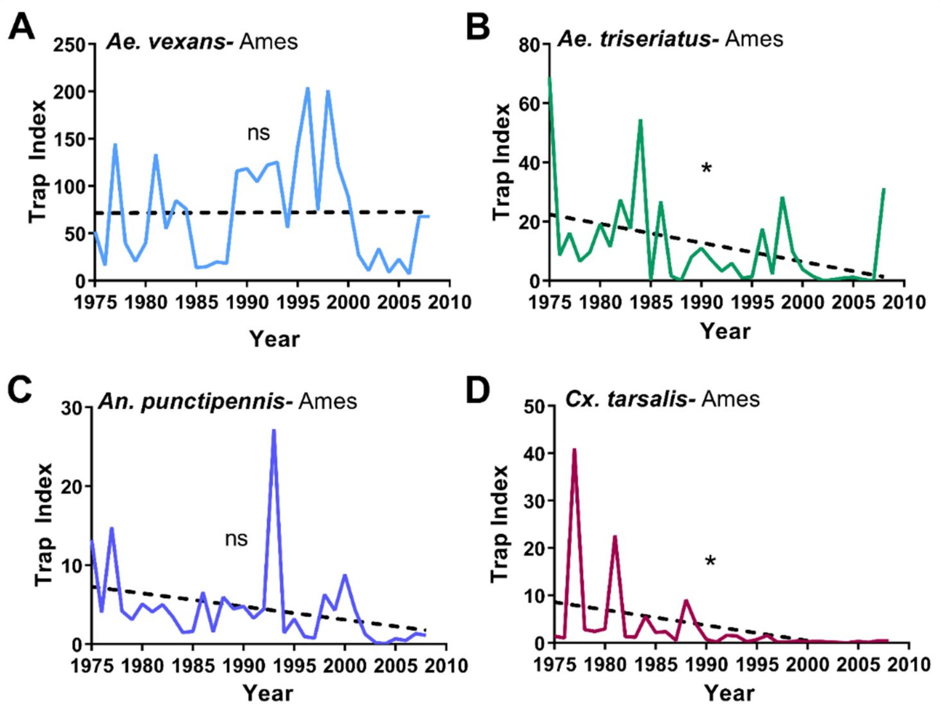

Figure S1. Comparisons of mosquito population abundance at the Ames study site. Trap indices for *Ae. vexans* (**A**), *Ae. triseriatus* (**B**), *An. punctipennis* (**C**), and *Cx. Tarsalis* (**D**) were examined over the study period and analyzed by simple linear regression to determine significance. The asterisk denotes significance (*= $P < 0.05$). ns, non-significant.

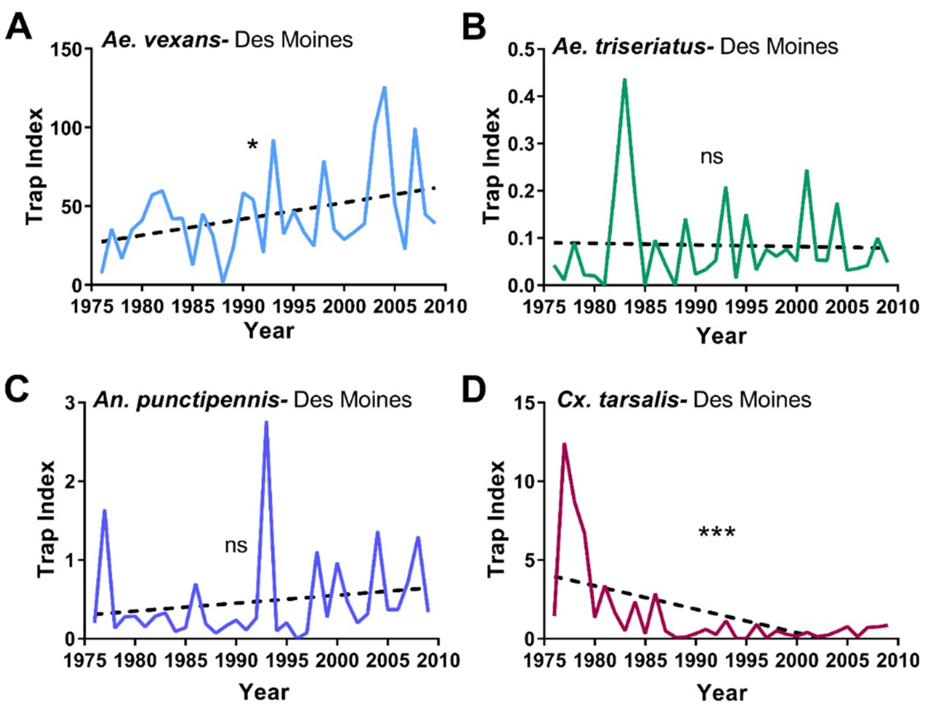

Figure S2. Comparisons of mosquito population abundance at the Des Moines study site. Trap indices for *Ae. vexans* (**A**), *Ae. triseriatus* (**B**), *An. punctipennis* (**C**), and *Cx. tarsalis* (**D**) were examined over the study period and analyzed by simple linear regression to determine significance. The asterisk denotes significance (*= $P < 0.05$; ***= $P < 0.001$). ns, non-significant.

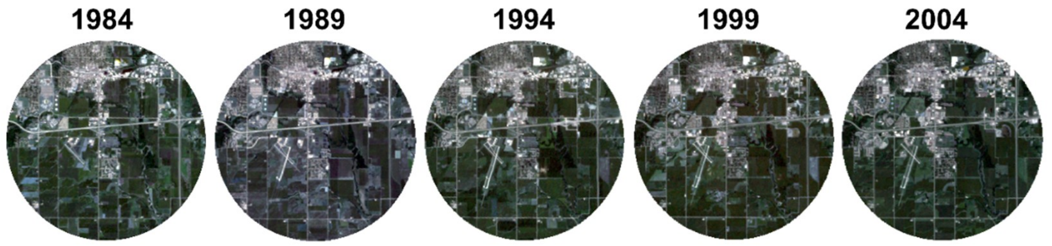

Figure S3. Unaltered Landsat 5 imagery of the Ames trapping site at five-year increments from 1984-2004. Satellite images centered over the trapping site were used for all land-use characterization. There are slight differences in the brightness of the photos due to atmospheric conditions at the time imagery was taken.

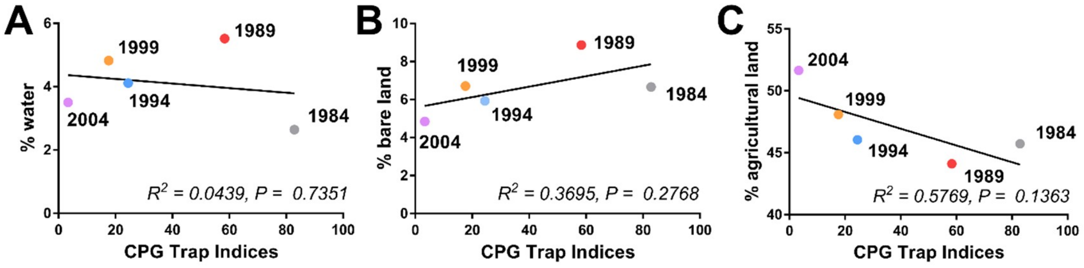

Figure S4. Influence of land use variables on *Culex pipiens* group (CPG) populations at the Ames trapping site. Relationships of CPG abundance (averaged over a five-year period ending in the year displayed) were examined by simple linear regression with the percentage of water (**A**), percent bare land (**B**), or agricultural land (**C**).

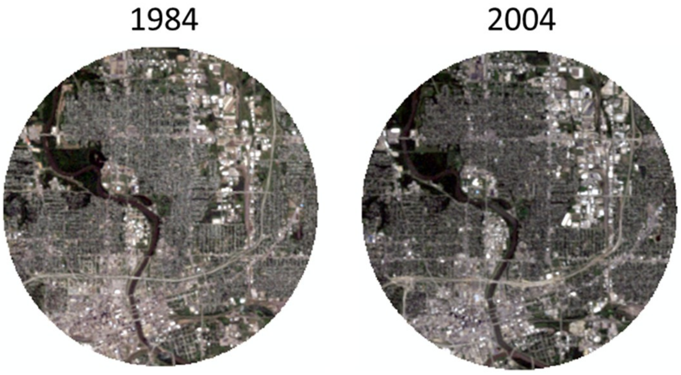

Figure S5. Unaltered Landsat 5 imagery of the Des Moines trapping site. Satellite images centered over the trapping site were used for all land-use characterization. There are slight differences in the brightness of the photos due to atmospheric conditions at the time imagery was taken.

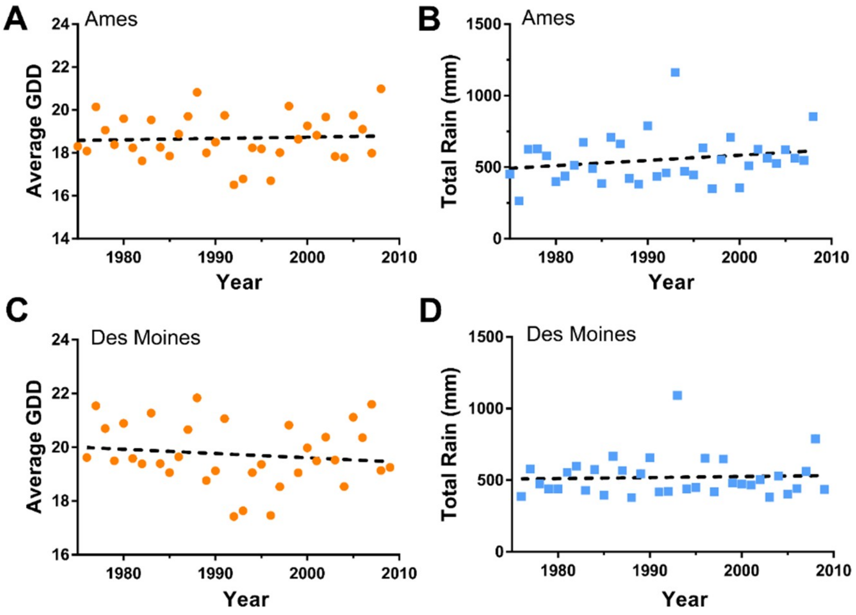

Figure S6. Climate variables do not significantly vary during the 34-year trapping period for both central Iowa sites. Average Growing Degree Day (temperature base 50 max 86) measurements (**A,C**) and total rainfall (mm) (**B,D**) are displayed from May 1st- October 1st of each given year for the Ames (**A,B**) or Des Moines (**C,D**) sites. Other than substantial flood events in 1993 that serve as a major outlier at both trapping locations, there are no significant differences in temperature or rainfall over time.

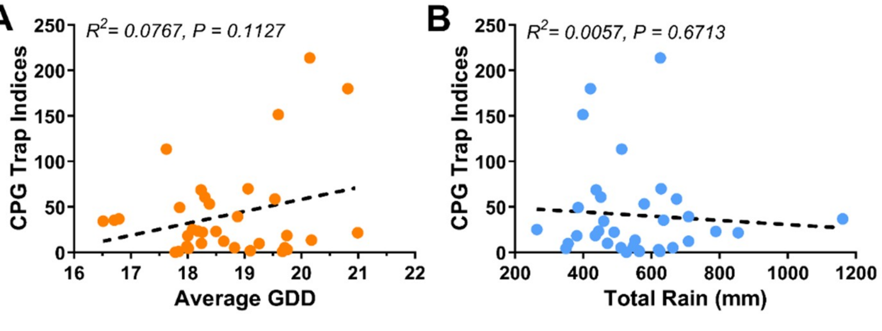

Figure S7. *Culex pipiens* group (CPG) abundance does not significantly correlate with temperature or rainfall. For the Ames trapping site, the relationship between CPG numbers (trap index) with temperature (**A**) or rainfall (**B**) were examined using simple linear regression analysis. No significant correlations with temperature or rainfall were determine during the study period.
